# Taraxerol Induces Cell Apoptosis through A Mitochondria-Mediated Pathway in HeLa Cells

**DOI:** 10.22074/cellj.2017.4543

**Published:** 2017-08-19

**Authors:** Xiangyang Yao, Binyu Lu, Chaotian Lü, Qin Bai, Dazhong Yan, Hui Xu

**Affiliations:** 1 Department of Biology and Food Engineering, Bengbu University, Bengbu, China; 2 School of Pharmacy, Fudan University, Shanghai, China; 3 School of Biology and Pharmaceutical Engineering, Wuhan Polytechnic University, Wuhan, China

**Keywords:** Taraxerol, Apoptosis, Mitochondria, HeLa Cells

## Abstract

**Objective:**

Taraxerol acetate has potent anti-cancer effects via the induction of apoptosis, autophagy, cell cycle arrest, and inhibition of cell migration. However, whether taraxerol induced apoptosis and its underlying mechanisms of action is not clear. In the present study, we assess the effects of taraxerol on the mitochondrial apoptotic pathway and determine the release of cytochrome c to the cytosol and activation of caspases.

**Materials and Methods:**

In this experimental study, we mainly investigated the effect of taraxerol on HeLa cells. We tested cell viability by the MTT assay and morphologic changes, analyzed apoptosis by DAPI staining and flow cytometry. We also determined reactive oxygen species (ROS) and mitochondrial membrane potential (MMP) using a Microplate Reader. In addition, the apoptotic proteins were tested by Western blot.

**Results:**

Taraxerol enhanced ROS levels and attenuated the MMP (Δψm) in HeLa cells. Taraxerol induced apoptosis mainly via the mitochondrial pathway including the release of cytochrome c to the cytosol and activation of caspases 9 and 3, and anti-poly (ADP- ribose) polymerase (PARP). Taraxerol could induce the down-regulation of the anti-apoptotic protein Bcl-2 and up-regulation of pro-apoptotic protein Bax. It suppressed the PI3K/ Akt signaling pathway.

**Conclusion:**

These results demonstrated that taraxerol induced cell apoptosis through a mitochondria-mediated pathway in HeLa cells. Thus, taraxerol might be a potential anticervical cancer candidate.

## Introduction

Cervical cancer is one of the most common malignant gynecological tumors in women worldwide. It is the third leading cause of cancer death among females in less developed countries (1). Concurrent radiotherapy with cisplatin-based chemotherapy plays a major role in treatment for early stage cervical cancer patients (2). However, patients who experience radiotherapy and concurrent chemoradiotherapy may also suffer from very strong adverse effects such as bowel obstruction, bleeding, urinary hematuria, or vaginal atrophy (3,4). Therefore, it is necessary to develop new drugs with less adverse effects for the treatment of cervical cancer. 

Taraxerol is a triterpenoid compound, which has potent anti-inflammatory and anti-cancer activities. Taraxerol has effectively inhibited NADPH oxidase (NOS) activity in murine microglial cells (5). Our previous study found that taraxerol significantly inhibited lipopolysaccharide (LPS)- induced production of interleukin-1 (IL-1β), IL- 6, and tumor necrosis factor-alpha (TNF-α) by interfering with the activation of TAK1 and Akt, thus preventing NF-κB activation (6). It remarkably inhibited TPA-induced tumor promotion on mouse spontaneous mammary tumors (7). Taraxerol has shown *in vitro* cytotoxic activity against HepG2 and A431 human cancer cell lines and potent inhibition of topoisomerase II (8). Taraxerol are also known to augment the inhibitory effects of cyclooxygenases-1 and -2 by measuring prostaglandin E2 (PGE2) production, and induce quinone reductase in cultured Hepa1c1c7 mouse hepatoma cells (9). Many triterpenoids exhibit potent anti-cancer effects via the induction of apoptosis in tumor cells, including HeLa (10,12). Recently, it has been reported that taraxerol acetate induced apoptosis, autophagy, cell cycle arrest, and cell migration in human glioblastoma cells and a mouse xenograft model (13). However, whether taraxerol induced apoptosis and its underlying mechanisms of action is not clear. In this study, we have used a human cervical cancer cell line (HeLa) to assess the effects of taraxerol on a mitochondrial apoptotic pathway and determined the release of cytochrome c to the cytosol, and the activation of caspases and anti-poly (ADP-ribose) polymerase (PARP). 

## Materials and Methods

### Antibodies and reagents

3-(4,5-Dimethyl-2-thiazolyl)-2,5-diphenyl-2H- tetrazolium bromide (MTT), dimethyl sulfoxide (DMSO), anti-Cox IV, anti-Bcl-2, anti-Bax, anti-cleaved caspase 3, anti-cleaved caspase 9, anti-cleaved caspase 8, PARP, anti-phospho-Akt (Ser473), anti-Akt antibodies and anti-cytochrome c were purchased from Cell Signaling Technology (Beverly, MA, USA). Anti-phospho-PI3K p85α (Tyr467), and anti-PI3K p85α antibodies were obtained from Santa Cruz Biotechnology (Santa Cruz, CA, USA). GAPDH was purchased from Bioworld Biotechnology. DAPI was obtained from Invitrogen (Grand Island, NY, USA). DMEM cell cultures and fetal bovine serum (FBS) were purchased from Gibco (Gaithersburg, MD, USA). Taraxerol was obtained from Sigma (St. Louis, MO, USA). 

### Cell culture and taraxerol treatment

We purchased human cervical cancer cells (HeLa) from the National Cell Bank of China, and maintained in DMEM medium supplemented with 10% FBS, 100 U/mL penicillin and 100 μg/ mL streptomycin. These cells were kept at 37˚C in a humidified atmosphere that contained 5% CO_2_. The taraxerol was dissolved in DMSO to make a stock of 100 mM and further diluted to final concentrations of 10-100 μM with a serum-free culture medium. The amount of DMSO added to the cell culture was less than 0.1% in all cases. This experimental study was approved by the Ethical Committee of Bengbu University.

### Cell viability assay

Cell viability was determined using the MTT assay. Briefly, cells were seeded in 96-well plates at 37˚C with 5% CO_2_ for overnight incubation and treated with appropriate concentrations of taraxerol for the indicated times. The cells were then incubated with a serum-free medium that contained MTT at a final concentration of 0.5 mg/ mL for 4 hours. The crystals formed in intact cells were solubilized in DMSO, and the absorbance was measured at 570 nm. Results were expressed as the percentages of reduced MTT, assuming the absorbance of control cells as 100%.

### Quantification of apoptotic cells by flow cytometry

The extent of apoptosis was measured by an annexin V-FITC/PI apoptosis detection kit (Invitrogen, CA, USA) according to the manufacturer’s instructions. Cells treated with taraxerol for 24 hours were harvested, washed twice with phosphate-buffered saline (PBS, Beyotime, China), gently re-suspended in binding buffer, and incubated with annexin-V-FITC and PI in the dark for 10 minutes, then detected by flow cytometry (BD Accuri C6). The data were analyzed with BD Accuri C6 Software. 

### DAPI staining assay

In this assay, HeLa cells were fixed with 4% paraformaldehyde for 10 minutes and stained with 1 μg/mL DAPI for 5 minutes, followed by observation under fluorescence microscope. 

### Mitochondrial membrane potential

Cells were pretreated different concentration of taraxerol for 8 hours, then the cells were stained with 10 μM of JC-1 (Beyotime) for 25 minutes at 37˚C. 

The cells were subsequently centrifuged and the staining solution removed. The cells were washed twice with JC-1 staining buffer. Fluorescence was monitored with an ELx 800 Universal Microplate Reader (Bio-Tek, Inc.) using 490/530 nm for the monomeric form and 525/590 nm for the aggregate of JC-1. We calculated the fluorescence intensity ratio of aggregates to monomers as an indicator of mitochondrial membrane potential (MMP). 

### Isolation of mitochondrial and cytosolic fractions

HeLa cells were suspended in a solution including (10 mM 4-(2-Hydroxyethyl)piperazine- 1-ethanesulfonic acid (HEPES, Beyotime, China), 10 mM KCl, 1 mM EDTA, 1 mM ethylene glycolbis(2-aminoethylether) tetraacetic acid (EGTA, Sigma, USA), 68 mM sucrose, 220 mM mannitol, 0.1% bovine serum albumin (BSA, Beyotime, China), and 1 mM phenylmethanesulfonyl fluoride (PMSF, Sigma, USA), 2 μg/mL aprotinin, and 0.1 mM leupeptin) for 30 minutes in ice to incubate. Cells were triturated and centrifuged at 200 x g for 2 minutes. We transferred the supernatant phase (500 μL) to a new tube, which was centrifuged at 7000 x g for 15 minutes to pellet all of the mitochondria. The supernatants were centrifuged at 16000 x g for 30 minutes and collected as cytosolic fractions. The mitochondrial fraction was subsequently solubilized in TBSTDS that consisted of 10 mM Tris (pH=7.5), 150 mM NaCl, 1 mM EDTA, 1% Triton X-100, 0.5% sodium deoxycholate (SDS), 0.02% NaN3, 0.0004% NaF and protease inhibitors. The above steps were carried out at 4˚C and then the fractions of mitochondria and cytosol were kept at -75˚C before use.

### Western blot analysis

After treatment with selected doses of taraxerol for the indicated times, HeLa cells were rinsed twice with ice-cold PBS, and lysed with the RIPA lysis buffer for 30 minutes on ice. Lysates were centrifuged (12000 x g) at 4˚C for 15 minutes. Total cell lysates (20 μg protein) were separated by SDS-polyacrylamide gel electrophoresis (PAGE) and transferred to nitrocellulose membranes or polyvinylidene difluoride membranes. Membranes were blocked for 60 minutes at room temperature. Membranes were probed with specific primary antibodies overnight at 4˚C and subsequently incubated with IRDye 800CW secondary antibodies for 1 hour. Membranes were visualized using an Odyssey Infrared Imaging Scanner. The fluorescence intensities were analyzed using Image Studio Software (Li-Cor Biosciences). 

### Statistical analysis

Data are presented as means ± SD from triplicate experiments. Statistical analyses were performed using an unpaired, two-tailed student’s t test. P<0.05 was considered significant. 

## Results

### Taraxerol reduced cell viability in HeLa cells

**Fig.1:**
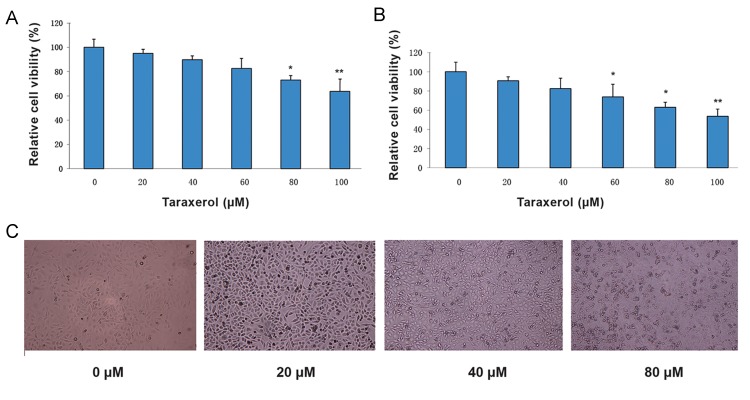
Effects of taraxerol on cell viability and morphological change in HeLa cells. A, B. HeLa cells were treated with different concentrations of taraxerol for 24 or 48 hours. Cell viability was examined by the MTT assay, and C. Morphological changes in HeLa cells after treatment with taraxerol for 24 hours as examined by phase-contrast microscopy. All images shown are representative of three independent experiments with similar results. *; P<0.05 and **; P<0.01 vs. control.

We investigated the effect of taraxerol on HeLa cell viability. Cells were treated with different concentrations of taraxerol for 24 or 48 hours. Cell viability was examined by the MTT assay. Cells treated with increasing concentrations of taraxerol for 24 hours had significantly reduced cell viability to 95% (20 μM), 89.8% (40 μM), 82.6% (60 μM), 72.9% (80 μM), and 63.6% (100 μM). Cells treated with taraxerol for 48 hours had cell viability of 90.7% at a concentration of 20 μM and 53.6% at the 100 μM concentration (Fig .1A,B). The effects of taraxerol on cell morphology were determined. The cells exposed to different dosages of taraxerol (0 to 80 μM) for 24 hours underwent significant morphological changes. As shown in Figure 1C, taraxerol treatment resulted in obvious cell loss and shrinkage, rounding and partial detachment. These results indicated that taraxerol could inhibit the growth of HeLa cells. 

### Taraxerol induced apoptosis in HeLa cells

**Fig.2:**
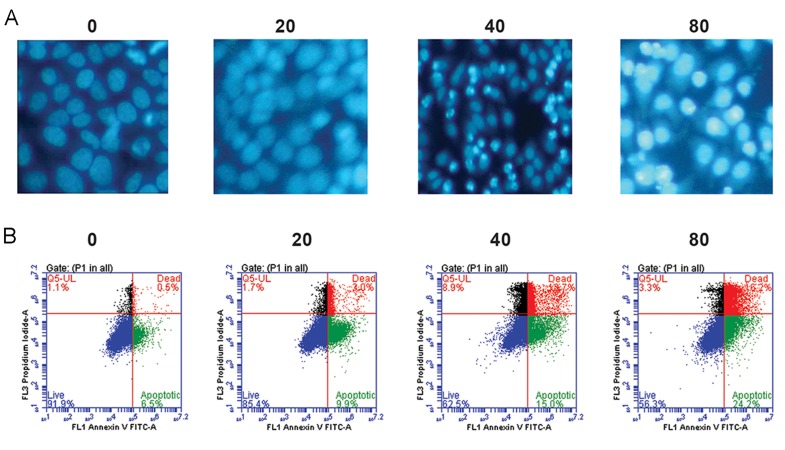
Taraxerol induced cell death in HeLa cells. The cells were treated with taraxerol at the indicated concentrations for 24 hours. A. DAPI stained cells were observed and photographed by fluorescence microscopy (magnification: ×200) and B. Percentage of apoptotic cells after treatment with various concentrations of taraxerol. Apoptosis was assessed by annexin V/PI double staining.

We sought to determine the occurrence of apoptotic cell death cause by taraxerol treatment. Cell morphological changes were examined by DAPI. We observed that, in a dose dependent manner, the chromatin became condensed and marginalized by pretreatment with different concentrations of taraxerol (Fig .2A). We further investigated apoptosis of cells by annexin V/ PI double staining. After incubation with 80 μM taraxerol for 24 hours, we observed a significant increase in both the early (24.2%) and late (16.2%) stages of apoptotic cells compared with the control group at the early (6.5%) and late (0.5%) stages (Fig .2B). These results demonstrated the ability of taraxerol to induce apoptosis in HeLa cells. 

### Taraxerol enhanced reactive oxygen species levels and attenuated the mitochondrial membrane potential in HeLa cells 

**Fig.3:**
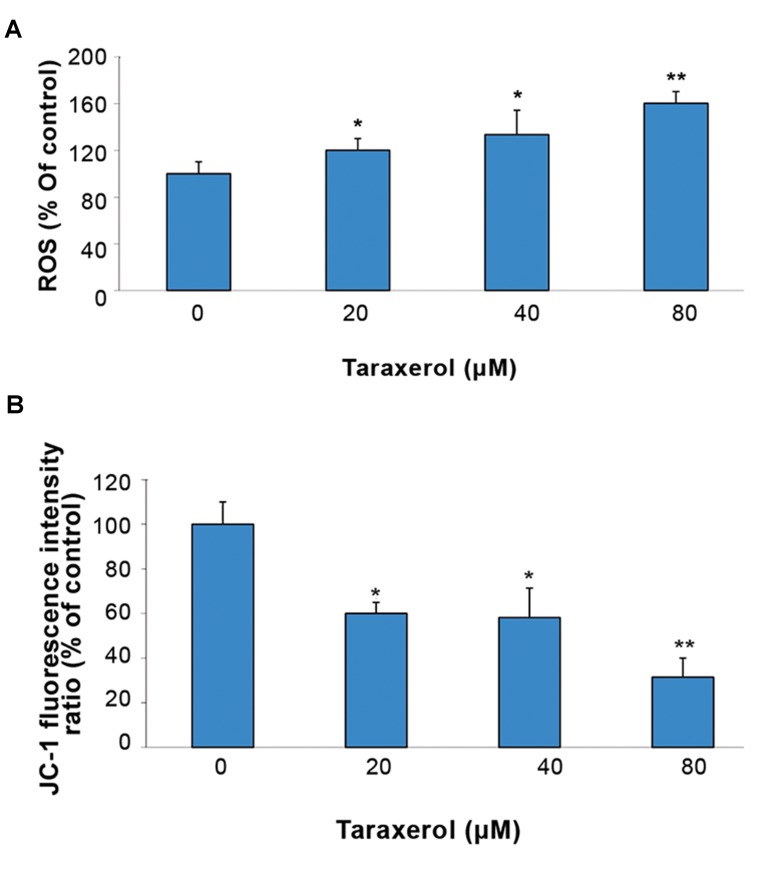
Effect of taraxerol on oxidative stress. Cells were incubated with or without taraxerol (20, 40, 80 μM) for 8 hours. A. Reactive oxygen species (ROS) were detected with DCFH2-DA and we calculated the fluorescence intensity and B. Mitochondrial membrane potential (MMP, Δψm) was evaluated by staining with JC-1 and analysis by a microplate reader. The quantitative Δψm from each group is expressed as the ratio of red (JC-1 aggregates)/green (JC-1 monomers). Data was expressed as a percentage of untreated controls. All experiments were repeated three times. The data are expressed as mean ± SD (n=3). *; P<0.05 and **; P<0.01 vs. control.

Intracellular reactive oxygen species (ROS) were detected using a DCFH-DA fluorescent probe. ROS production increased 33.4% with 40 μM of taraxerol and 60.2% with 80 μM of taraxerol compared to the control (Fig .3A). Δψm was analyzed by JC-1 fluorescence. Taraxerol-treated cells displayed a loss of MMP (69%, Fig .3B). Taraxerol promoted the breakdown of MMP by preservation of the red JC-1 fluorescence. 

### Effect of taraxerol on cytochrome c, Bcl-2 and Bax expression in HeLa cells

**Fig.4:**
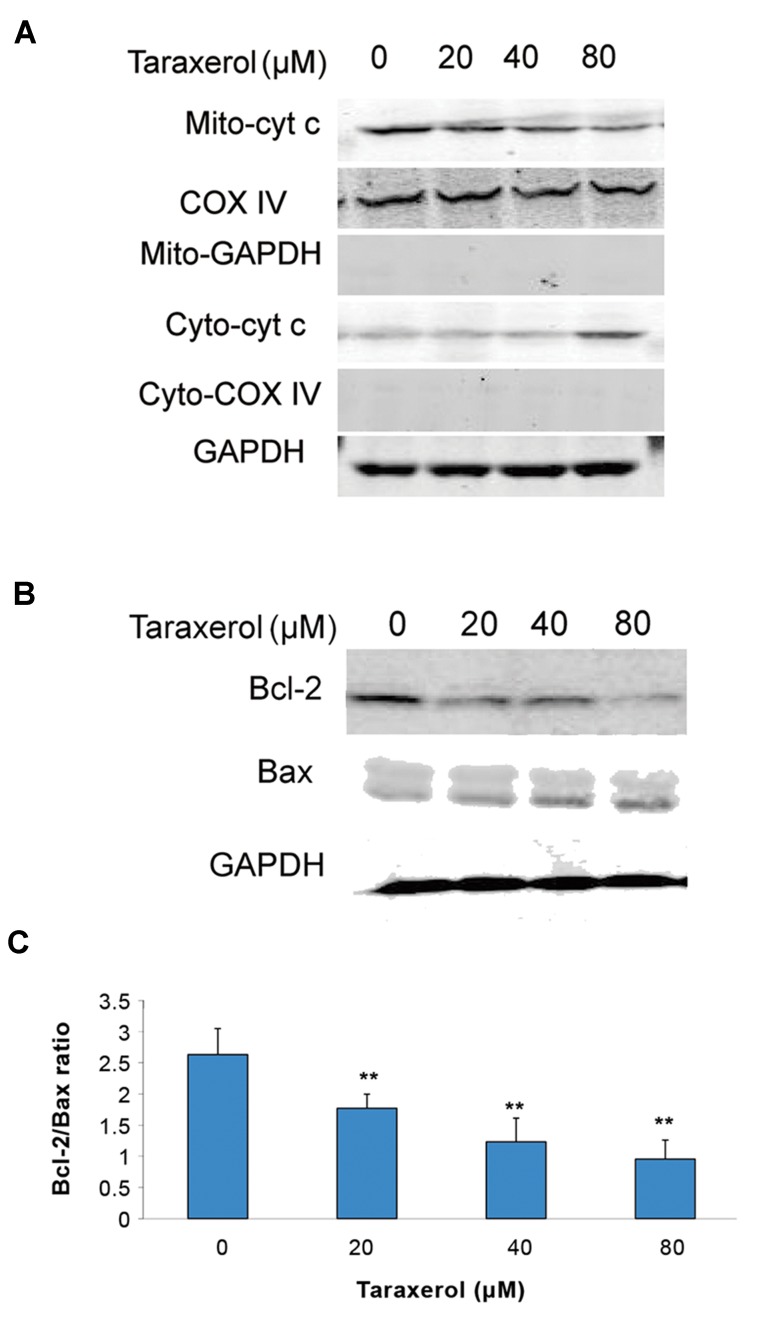
Effect of taraxerol on cytochrome c release, Bax, and Bcl-2. A. Cells were incubated with taraxerol (0, 20, 40, 80 μM) for 12 hours. Expression of mitochondrial cytochrome c (Mito-cyto c), cytosolic cytochrome c (Cyto-cyto c). Cox IV and GAPDH were as control in the mitochondrial and cytosolic fractions, respectively, B. Expressions of Bax and Bcl-2 as measured by Western blot, and C. The ratio of Bcl-2/Bax. All experiments were repeated three times. The data are expressed as mean ± SD (n=3). **; P<0.01 vs. control.

Next, we assessed the potential involvement of inhibition of cytochrome c release. We found that the cytochrome c levels markedly decreased in the cytosolic fraction and increased in the mitochondrial fraction with the taraxerol-treated group (Fig .4A). Furthermore, Bcl-2 and Bax were determined. The data demonstrated that taraxerol could induce downregulation of the anti-apoptotic protein Bcl-2 and upregulation of the pro-apoptotic protein Bax. The decreased ratio of Bcl-2/Bax could exacerbate cytochrome c release, thereby increasing apoptosis (Fig .4B,C). 

### Effect of taraxerol on caspases 8, 9, and 3, and anti-poly (ADP-ribose) polymerase expression in HeLa cells 

**Fig.5:**
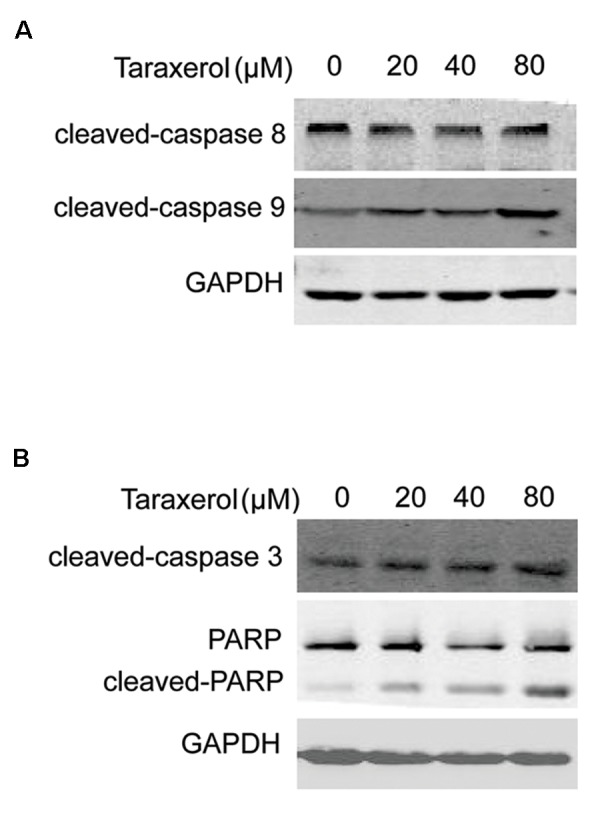
Taraxerol alters the expression of caspases 8, 9, and 3, and cleavage of anti-poly (ADP-ribose) polymerase (PARP). Cells were incubated with taraxerol (0, 20, 40, 80 μM) for 12 hours. A. The expressions of caspase 8 and 9 as assessed by immunoblotting and B. Expressions of Bax and Bcl-2, cleaved caspase 8 and cleaved caspase 9 as measured by Western Blot. GAPDH was used as the loading control. The results were expressed as mean ± SD of three independent experiments.

We assessed caspases 8 and 9 to understand the mechanism of taraxerol-induced apoptosis. The results demonstrated that caspase 9 activity obviously increased, while caspase 8 was unchanged in the HeLa cells (Fig .5A). Our data also showed that taraxerol increased the levels of activated caspase 3 and cleaved PARP in a concentration-dependent manner in these cells. spase 3 and PARP, as the main cleavage targets of effector caspases 8 and 9, suggested that taraxerol induced apoptosis via the mitochondrial pathway including the release of cytochrome c to the cytosol and the activation of caspases 9, caspases 3 and PARP (Fig .5B). 

### Taraxerol decreased the PI3K/Akt signaling pathway

**Fig.6:**
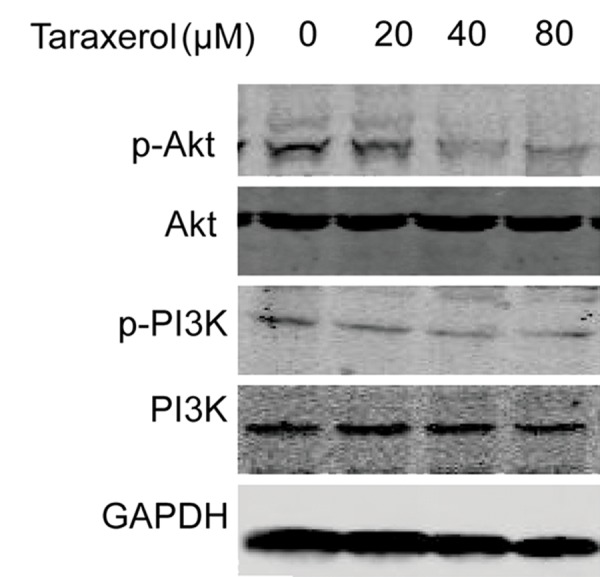
Effects of taraxerol on phosphorylation of PI3K/Akt in HeLa cells. Cells were pretreated with 0, 20, 40, or 80 μM of taraxerol for 6 hours. The cell lysates were collected and analyzed by Western blot with anti-phosphorylated PI3K and anti-phosphorylated Akt. The results were expressed as mean ± SD of three independent experiments.

In our previous study, taraxerol reduced Akt activation in LPS-induced RAW264.7 cells (6). We explored the roles of these signaling pathways in taraxerol-induced apoptosis by determining the total and phosphorylated levels of Akt and PI3K. Western blot analysis showed that the protein levels of p-PI3K and p-Akt both reduced in a dose-dependent manner. Total Akt and PI3K levels remained unchanged (Fig .6). Collectively, these results demonstrated that taraxerol might induce cell apoptosis via decreased PI3K/Akt signaling. 

## Discussion

In this study, taraxerol modulated apoptosis in HeLa cells via morphological changes, cell viability, activation of caspase, and PARP. DAPI staining and flow cytometry revealed that both apoptotic bodies and the percentage of apoptotic cells significantly increased in a dose-dependent manner. These results indicated that taraxerol exhibited anti-cancer activity through induction of apoptosis. It has been reported that mitochondrial outer membrane permeabilization (MOMP) mediated the release of cytochrome c from mitochondria in the mitochondrial pathway (the intrinsic pathway). Cytochrome c binds with Apaf- 1 and caspase 9 to form the apoptosome, which cleave and activate caspases 3 and 7, subsequently leading to cell apoptosis. Our data have indicated that taraxerol caused the release of cytochrome c and activation of caspases 9 and 3 in HeLa cells. Furthermore, mitochondrial damage plays an important role in the apoptosis process. The loss of MMP (Δψm) and an increase in ROS production are crucial parameters of mitochondrial damage. In this study, we have observed that taraxerol enhanced ROS levels and the MMP (Δψm) in HeLa cells. 

The ratio of Bcl-2 to Bax appears to be one of the critical factors of cell apoptosis (14). Thus, downregulation of the Bcl-2/Bax expression ratio could be a predominant mitochondria-mediated apoptosis mechanism. Our current results have demonstrated that taraxerol treatment led to a significant loss of MMP, downregulated Bcl-2 expression, and increased Bax levels in HeLa cells which suggested that Bcl-2 and Bax participated in controlling taraxerol-induced mitochondrial damage. However, we could not rule out the possibility that other Bcl-2 family proteins might be involved. PI3K/Akt signaling plays a critical role in prevention of apoptosis (15). The PI3K/ Akt pathway has been shown to inhibit Bax translocation from cytoplasm to the mitochondria and upregulate Bcl-2 expression (16,17). Consistent with our previous study, we showed that the protein level of p-Akt reduced in a dose- dependent manner. Recent studies demonstrated that natural or synthetic triterpenoids inhibited the Akt pathways, which decreased Bcl-2/Bax ratio in cells (18,19). Blockage of PI3K/Akt signaling promotes apoptosis in neoplastic cells. Thus, it is possible that suppression of PI3K and Akt activation is a critical target for inhibition of Bcl-2 by taraxerol. 

## Conclusion

The results demonstrated that taraxerol could significantly reduce HeLa cell survival and induce apoptosis mainly via the mitochondrial pathway which included the release of cytochrome c to the cytosol and activation of caspases. Taraxerol suppressed the PI3K/Akt signaling pathway. Thus, taraxerol might be a potential anticancer drug candidate. 

## References

[1] Torre LA, Bray F, Siegel RL, Ferlay J, Lortet-Tieulent J, Jemal A (2015). Global cancer statistics, 2012. CA Cancer J Clin.

[2] Zhao H, Li L, Su H, Lin B, Zhang X, Xue S (2016). Concurrent paclitaxel/cisplatin chemoradiotherapy with or without consolidation chemotherapy in high-risk early-stage cervical cancer patients following radical hysterectomy: preliminary results of a phase III randomized study. Oncotarget.

[3] Green JA, Kirwan JM, Tierney JF, Symonds P, Fresco L, Collingwood M (2001). Survival and recurrence after concomitant chemotherapy and radiotherapy for cancer of the uterine cervix: a systematic review and meta-analysis. Lancet.

[4] Jensen PT, Klee MC, Groenvold M (2002). Validation of a questionnaire for self-rating of urological and gynaecological morbidity after treatment of gynaecological cancer. Radiother Oncol.

[5] Tsao CC, Shen YC, Su CR, Li CY, Liou MJ, Dung NX (2008). New diterpenoids and the bioactivity of Erythrophleum fordii. Bioorg Med Chem.

[6] Yao X, Li G, Bai Q, Xu H, Lü C (2013). Taraxerol inhibits LPSinduced inflammatory responses through suppression of TAK1 and Akt activation. Int Immunopharmacol.

[7] Takasaki M, Konoshima T, Tokuda H, Masuda K, Arai Y, Shiojima K (1999). Anti-carcinogenic activity of Taraxacum plant.II. Biol Pharm Bull.

[8] Setzer WN, Shen X, Bates RB, Burns JR, McClure KJ, Zhang P (2000). A phytochemical investigation of Alchornea latifolia. Fitoterapia.

[9] Jang DS, Cuendet M, Pawlus AD, Kardono LB, Kawanishi K, Farnsworth NR (2004). Potential cancer chemopreventive constituents of the leaves of Macaranga triloba. Phytochemistry.

[10] Tang J, Xu J, Zhang J, Liu WY, Xie N, Chen L (2016). Novel tirucallane triterpenoids from the stem bark of Toona sinensis. Fitoterapia.

[11] Ruan W, Wei Y, Popovich DG (2015). Distinct responses of cytotoxic ganoderma iucidum triterpenoids in human carcinoma cells. Phytother Res.

[12] Farimani MM, Abbas-Mohammadi M, Esmaeili MA, Salehi P, Nejad-Ebrahimi S, Sonboli A (2015). Seco-ursane-type triterpenoids from Salvia urmiensis with apoptosis-inducing activity. Planta Med.

[13] Hong JF, Song YF, Liu Z, Zheng ZC, Chen HJ, Wang SS (2016). Anticancer activity of taraxerol acetate in human glioblastoma cells and a mouse xenograft model via induction of autophagy and apoptotic cell death, cell cycle arrest and inhibition of cell migration. Mol Med Rep.

[14] Cory S, Adams JM (2005). Killing cancer cells by flipping the Bcl- 2/Bax switch. Cancer Cell.

[15] Vanhaesebroeck B, Stephens L, Hawkins P (2012). PI3K signalling: the path to discovery and understanding. Nat Rev Mol Cell Biol.

[16] Pugazhenthi S, Nesterova A, Sable C, Heidenreich KA, Boxer LM, Heasley LE (2000). Akt/protein kinase B upregulates Bcl-2 expression through cAMP-response element- binding protein. J Biol Chem.

[17] Tsuruta F, Masuyama N, Gotoh Y (2002). The phosphatidylinositol 3-kinase (PI3K)-Akt pathway suppresses Bax translocation to mitochondria. J Biol Chem.

[18] To C, Ringelberg CS, Royce DB, Williams CR, Risingsong R, Sporn MB (2015). Dimethyl fumarate and the oleanane triterpenoids, CDDO-imidazolide and CDDO-methyl ester, both activate the Nrf2 pathway but have opposite effects in the A/J model of lung carcinogenesis. Carcinogenesis.

[19] Hsu RJ, Hsu YC, Chen SP, Fu CL, Yu JC, Chang FW (2015). The triterpenoids of Hibiscus syriacus induce apoptosis and inhibit cell migration in breast cancer cells. BMC Complement Altern Med.

